# Enhancing Hypertension Risk Diagnosis Using a Hybrid Machine Learning Framework: Leveraging Body Composition Data

**DOI:** 10.1155/bmri/6335947

**Published:** 2026-02-01

**Authors:** Abdul Wahid Mirzaye, Hamid Saadatfar, Mohammad Ali Nematollahi

**Affiliations:** ^1^ Department of Computer Engineering, Faculty of Electrical and Computer Engineering, University of Birjand, Birjand, Iran, birjand.ac.ir; ^2^ Department of Computer Sciences, Fasa University, Fasa, Iran

**Keywords:** body composition data, Gaussian naive Bayes, hypertension diagnosis, K-Means clustering, random search optimization, SMOTE technique

## Abstract

Hypertension, widely recognized as the “silent killer,” remains a leading cause of cardiovascular, renal, and neurological complications worldwide. This study proposes a dual‐scenario hybrid machine learning framework for hypertension risk prediction using noninvasive body composition features, aimed at enhancing both interpretability and predictive reliability. In Scenario 1, an unsupervised clustering analysis inspired by self‐labeling principles was performed exclusively on hypertensive individuals, where five physiological subgroups were identified via K‐Means clustering and validated using Silhouette (0.3371), Davies–Bouldin (1.0094), and Calinski–Harabasz (720.10) indices. Significant intercluster variability (*p* < 0.001) was observed across key indicators such as FATP, RLFATP, LLFATP, FATM, and age. Among the tested models, the support vector machine (SVM) with random oversampling achieved the best performance (accuracy = 99.08*%*, F1 = 98.04*%*, AUC = 99.98*%*), confirming effective subgroup discrimination. In Scenario 2, a comprehensive binary classification between healthy and hypertensive subjects was conducted using five models—ExtraTrees, KNN, SVM, Gaussian Naive Bayes, and Decision Tree—across multiple configurations. The cluster‐augmented dataset yielded the best results, with the ExtraTrees classifier achieving superior performance (accuracy = 98.23*%*, recall = 98.30*%*, precision = 98.17*%*, F1 = 98.23*%*, AUC = 99.87*%*). Clustering and feature selection both improved generalization, particularly for ensemble‐based learners. Overall, Scenario 2 demonstrated the highest predictive accuracy and stability, whereas Scenario 1 provided valuable interpretability through subgroup discovery. These findings highlight that integrating unsupervised clustering with supervised classification offers a robust and explainable framework for personalized hypertension risk prediction, contributing to early detection and precision healthcare.

## 1. Introduction

Hypertension, or high blood pressure (BP), is a major global health issue that causes significant illness, death, and economic difficulties [1]. Often called a “silent killer,” hypertension usually does not present any symptoms, making it a leading cause of cardiovascular diseases (CVDs). Serious complications such as strokes, heart attacks, and kidney disease often occur before people realize they have it. In 2010, around 1.33 billion people worldwide had hypertension, which is about 19.3% of the global population. This number is expected to rise to 1.56 billion by 2025, which shows the urgent need for effective and easy‐to‐use early detection and management methods. This problem is especially serious in low‐income and middle‐income countries, where limited healthcare resources put extra stress on already strained healthcare systems, making it harder to manage chronic diseases [2].

Many factors contribute to the prevalence of hypertension. These include an aging population, changes in lifestyle, and genetic factors. Certain lifestyle choices, such as obesity, high salt intake, drinking too much alcohol, and not getting enough exercise, can significantly raise BP, especially in busy and high‐stress areas [3]. Environmental factors also play a role [4]. Pollutants like heavy metals—including cadmium, mercury, and selenium—can increase the risk of hypertension, particularly in industrialized or polluted areas. A study by Miao et al. highlighted that long‐term exposure to chemicals like cadmium and certain heavy metals, such as vanadium and iron, can worsen the risk of developing high BP [5].

Hypertension has serious economic effects. In the United States, healthcare costs related to hypertension were about $46 billion in 2011. This amount is expected to rise sharply to $274 billion by 2030 due to an aging population and the growing number of people with hypertension. Worldwide, hypertension and its complications make up 10% of total healthcare costs, showing how much of an impact this condition has [6]. Although doctors can manage hypertension with medication and lifestyle changes, the high number of people with this condition and its often‐hidden symptoms mean that we need better ways to find and predict hypertension. This is important to prevent serious health issues.

Recent advancements in computer‐aided decision‐making, that is, machine learning (ML), have offered new ways to address complex healthcare issues like hypertension [7] ML allows computers to learn from data and make diagnoses without being specifically programmed. It shows great promise in healthcare for predicting diseases, assessing risks, and managing patient care.

Many large datasets, such as electronic health records (EHRs), environmental data, and personal health records, provide valuable information. ML models can analyze this data to find hidden patterns related to the risk of hypertension. Unlike traditional statistical methods, ML can handle complex and large amounts of data. This capability helps researchers understand how genetic, lifestyle, and environmental factors interact to affect hypertension.

Several ML techniques have proven effective in predicting hypertension. logistic regression (LR) models, which categorize data based on historical trends, are frequently used to predict hypertension as a binary outcome [8]. Advanced tree‐based algorithms, such as extreme gradient boosting (XGBoost), have demonstrated particularly high accuracy in hypertension risk diagnosis, with AUROC (area under the receiver operating characteristic) scores of 0.87 and 0.88, respectively, when predicting hypertension over 2 years [9].

In addition to predictive modeling, wearable health devices have enhanced ML′s application in hypertension management by providing real‐time data on vital health metrics, such as heart rate, BP, and physical activity levels [10]. These devices allow continuous monitoring, which is essential for a condition like hypertension that requires frequent assessment. Regular monitoring through wearables, coupled with ML‐powered analytics, enables early detection of abnormal BP patterns, facilitating timely intervention and improving patient outcomes [11, 12]. This approach aligns with the broader shift towards personalized medicine, wherein treatment plans are customized based on individual health profiles.

ML′s applicability is not limited to adult populations; studies have also shown its potential in predicting hypertension risk among adolescents and young adults. For example, in Malaysia, 24.5% of adolescents reportedly suffer from elevated BP, a statistic that reflects the increasing incidence of hypertension among younger populations [13]. By leveraging easily obtainable data, such as body mass index (BMI), waist circumference, and lifestyle behaviors, ML models can identify at‐risk individuals and inform preventive interventions at an early stage. The ability to predict hypertension risk across age groups and demographics highlights the versatility of ML models in supporting public health efforts aimed at combating hypertension.

Hence, this research is aimed at developing a two‐scenario hybrid ML framework for hypertension diagnosis using noninvasive body composition data. In the first scenario, an unsupervised clustering analysis, conceptually inspired by self‐labeling principles, was performed exclusively on hypertensive individuals (*n* = 1,099). The K‐Means algorithm was then applied to identify latent physiological subgroups within this population.

[14, 15]. The resulting five clusters were validated through Silhouette = 0.3371, Davies–Bouldin = 1.0094, and Calinski–Harabasz = 720.10, indicating robust cluster quality. Significant intercluster variability (*p* < 0.001) was observed across critical indicators such as fat percentage (FATP), RLFATP, LLFATP, fat mass (FATM), and age, enhancing the interpretability of hypertension subtypes.

In the second scenario, a supervised binary classification (healthy vs. hypertensive) is performed using five models—ExtraTrees, KNN, support vector machine (SVM), Gaussian Naive Bayes (GNB), and Decision Tree—across three configurations. The best overall performance was achieved using ExtraTrees, with accuracy = 98.23*%*, recall = 98.30*%*, precision = 98.17*%*, F1 = 98.23*%*, and area under the curve (AUC) = 99.87*%*, whereas SVM with random oversampling demonstrated superior cluster‐level discrimination with accuracy = 99.08*%*, F1 = 98.04*%*, and AUC = 99.98*%*.

Normalization and the synthetic minority oversampling technique (SMOTE) were applied to handle class imbalance, and random search optimization was employed for hyperparameter tuning [16]. The use of 10‐fold cross‐validation ensured robust and generalizable results [17]. Overall, the proposed dual‐scenario design provides both interpretability through patient subgroup discovery and diagnostic reliability through classification accuracy.

The main contributions of this research are as follows:

1. Dual‐scenario hybrid framework: introduction of a two‐scenario approach combining unsupervised clustering and supervised classification. Scenario 1 achieved strong clustering validity (Silhouette = 0.3371, Davies–Bouldin = 1.0094, Calinski–Harabasz = 720.10), enabling meaningful subgroup discovery among hypertensive individuals. Scenario 2 achieved high diagnostic performance with ExtraTrees (accuracy = 98.23*%*, recall = 98.30*%*, F1 = 98.23*%*, AUC = 99.87*%*), demonstrating the framework′s predictive strength.
2. Interpretability through unsupervised learning: application of K‐Means clustering to reveal physiological subgroups and intercluster differences (*p* < 0.001) across key body composition features (FATP, RLFATP, LLFATP, FATM, and age), improving model transparency and clinical insight.
3. Body composition‐based predictive modeling: utilization of noninvasive body composition indicators for hypertension risk assessment, providing a cost‐effective and scalable diagnostic strategy compared with biochemical testing.
4. Performance robustness via data balancing and optimization: implementation of SMOTE to address class imbalance and random search optimization for hyperparameter tuning, resulting in high accuracy and generalization stability across all tested models.
5. Clinical impact and explainability: demonstration that integrating clustering‐driven interpretability with supervised learning improves both diagnostic reliability and patient‐level explainability, supporting personalized hypertension risk prediction in clinical practice.

The remainder of this paper is organized as follows: Section 2 reviews the related works, Section 3 describes the study population, Section 4 presents the materials and methods, Section 5 discusses the experimental results and comparative analysis, and Section 6 concludes the paper with key findings and directions for future work.

## 2. Related Works

A growing number of research focuses on predicting the development and effects of hypertension. Currently, most hypertension diagnosis models are created utilizing ML methodologies. Liu et al. investigated the relationship between ambient chemical exposure and hypertension using NHANES (2005–2012) data and ML models [2]. The study comprised 4,492 subjects, and 29 important variables (21 chemicals) were discovered using feature selection and correlation analysis. Five ML models were used: SVM, XGBoost, random forest (RF), LR, and multilayer perceptron (MLP). SVM has the greatest AUC (0.731), followed by XGBoost (0.729). Amaratunga et al. investigated the use of ML in hypertension research, with an emphasis on its predictive capacities for cardiovascular risk and illness treatment [18]. They looked at several ML methodologies, such as deep learning, neural networks (NNs), and SVM. In one case, deep learning and SVM predicted cardiovascular events with 56%–57% accuracy. Another NN research, using data from 378,256 patients, found 68% sensitivity, 71% specificity, and an AUC of 0.87 for predicting 10‐year cardiovascular outcomes. Furthermore, an ML model examining 1.5 million patients identified hypertension with 51% sensitivity, 99% specificity, and an AUC of 0.87. Zhao et al. investigated ML‐based models for predicting secondary hypertension in diabetic patients [19]. The study analyzed data from 2,080 patients at Qingdao University′s metabolic illness clinic using five ML algorithms: artificial neural network (ANN), decision tree, RF, SVM, and Bayesian network. predictions were made using noninvasive variables such as demographics, physical measurements, and everyday living circumstances. Among the models examined, ANN performed the best, with an accuracy of 92.47%, sensitivity of 92.98%, specificity of 92.02%, and an AUC of 0.951 on the testing dataset.

Hoffman et al. created an ML model to predict postpartum readmission owing to hypertensive disorders of pregnancy (HDP) within 42 days [20]. Using data from 25,559 births, XGBoost was applied to 31 clinical variables, including systolic BP trends, preeclampsia, and new factors such as ketorolac usage. The model had AUCs of 0.85 and 0.81 in the derivation and validation cohorts, respectively. Lopez‐Martinez et al. created a LR model to predict hypertension using data from 19,709 NHANES participants (2007–2016) [6]. The study assessed noninvasive risk factors such as age, gender, BMI, race, smoking habits, renal disease, and diabetes using transformed categorical variables to guarantee uniformity. The model has an AUC of 0.73 (95% confidence interval: 0.70–0.76), 77% sensitivity, and 68% specificity. D′Silva et al. created a machine learning model to predict hypertension using ambulatory blood pressure monitor (ABPM) data [21]. The model used supervised learning and a decision tree classifier to identify BP conditions as normal, high, hypertensive risk, or severe hypertension. The training dataset, which included ABPM measurements from 1,516 patients, was structured into labeled.csv files, allowing the model to learn patterns of BP fluctuation. The decision tree classifier was effective, as evidenced by a confusion matrix and a low root mean square error of 0.0512, suggesting good accuracy. Chang et al. developed a ML‐based technique for predicting hypertension outcomes using physical examination data [22]. Their technique consists of two steps: feature selection using recursive feature elimination combined with cross‐validation to find the most relevant predictors and result in diagnosis utilizing classification algorithms such as SVM, C4.5 decision tree, RF, and XGBoost. Among them, XGBoost performed the best, with an accuracy of 94.36%, an F1 score of 0.875, and an AUC of 0.927 utilizing only a few characteristics, such as BP indicators. Montagna et al. studied ML techniques to improve hypertension identification using data acquired from World Hypertension Day campaigns (2015–2019), which included 20,206 participants [23]. The study examined five ML algorithms: LR, decision trees, RFs, SVM, and XGBoost. They also addressed class imbalance by employing oversampling (SMOTE) and undersampling techniques. RFs achieved the best balance, with a sensitivity of 0.818, specificity of 0.629, accuracy of 0.681, and AUC of 0.816 using an undersampling strategy. XGBoost has the best sensitivity (0.988), but low specificity (0.113).

Because body composition data were used to feed the ML algorithm in the current study, a few studies have focused on hypertension using this sort of data. Nematollahi et al. used 15 algorithms to predict hypertension using body composition data [24]. The dataset consisted of 1,099 hypertensive patient samples from a population‐based investigation. Their findings revealed that the AutoMLP, stacking, and voting approaches performed the best in predicting hypertension, with accuracies of 90%, 84%, and 83%, respectively. In another study, Seo et al. presented ML models to predict hypertension in Korean people by analyzing body composition variables such as body fat mass (BFM), skeletal muscle mass (SMM), and total body water (TBW) [25]. They tested six algorithms, including elastic net (E‐net), RF, XGBoost, NN, k‐nearest neighbor (K‐NN), and SVM. The E‐net model produced the greatest AUC (0.865 for males and 0.831 for females). Despite substantial advances in state‐of‐the‐art (SOTA) ML and deep learning models for hypertension prediction, several methodological gaps remain that directly motivate our choice of a hybrid clustering–classification framework. Most supervised models in the literature learn a single global decision boundary, which makes them highly dependent on large, labeled datasets and limits their robustness when applied to heterogeneous clinical populations. This limitation is particularly important for hypertension, where patients with similar diagnostic labels may exhibit markedly different physiological patterns. To overcome this, our framework first incorporates unsupervised clustering to explicitly reveal latent subgroups that conventional ML/DL models tend to overlook.

In addition, deep learning approaches—although effective in extracting complex representations—usually operate as black‐box systems, offering little transparency regarding the physiological factors contributing to risk. By using clustering followed by supervised modeling, our method provides a structured representation of subgroup characteristics that enhances interpretability and aligns better with clinical reasoning.

Another challenge repeatedly reported in SOTA studies is the difficulty of handling class imbalance, feature interaction ambiguity, and overfitting on high‐dimensional inputs. In our design, the separation of exploratory subgroup discovery from supervised prediction helps reduce these vulnerabilities by allowing the classifier to operate within more coherent and physiologically meaningful partitions of the data.

Collectively, this hybrid two‐scenario formulation addresses limitations that standalone ML/DL models cannot easily resolve: It improves generalization across heterogeneous populations, uncovers previously hidden physiological structures, and offers a more interpretable diagnostic pathway while relying solely on noninvasive clinical indicators.

Although ML has been widely used for hypertension prediction, much of the existing literature still depends on single‐scenario supervised models. Such approaches provide limited room for exploring the underlying structure of the data and, as a result, are not well suited for uncovering latent physiological subgroups. Our study responds to this gap by adopting a dual‐scenario framework that brings together unsupervised clustering and supervised classification in a coherent design (Contribution 1). Another challenge in earlier work is the restricted interpretability of many models, which often behave like black boxes with little indication of the physiological distinctions that drive their predictions. In contrast, by applying K‐Means alongside a statistical comparison of key body composition variables, we were able to highlight meaningful intercluster patterns and offer a more transparent view of model behavior (Contribution 2).

A further limitation in prior studies is their frequent reliance on biochemical measurements. Although these markers are useful, they tend to increase cost and limit the practicality of large‐scale deployment, particularly in settings with limited resources. By building our predictive framework around noninvasive body composition indicators, we provide an alternative that is both affordable and easier to implement in routine screening (Contribution 3). Additionally, several published models have not adequately addressed class imbalance or carried out systematic hyperparameter tuning—two factors that can substantially weaken generalization. Incorporating SMOTE and randomized optimization helped us achieve more stable and balanced performance across different algorithms (Contribution 4).

Finally, earlier research seldom brought together exploratory subgroup analysis and supervised diagnostic modeling in a way that could support individualized interpretation. The integrated nature of our approach makes it possible to deliver both strong predictive accuracy and personalized, physiologically meaningful risk profiles (Contribution 5). Taken together, these elements close several of the key gaps noted in previous machine learning studies on hypertension and enable forms of analysis that were not feasible in earlier work.

## 3. Study Population

Our study dataset comprised records from a subset of participants in an observational cohort study conducted in Fasa, a city of approximately 250,000 residents in Fars Province, Iran. This dataset was originally collected to investigate associations between risk factors and noncommunicable diseases among rural residents [26, 27]. We included 4,663 subjects (mean age 47.64 ± 9.37 years, range 35–70 years, and 2,156 males and 2,507 females). Informed consent was obtained from all participants and from legal guardians where applicable. Permission from the authors of the original study was obtained for use of the dataset, along with institutional approval for retrospective analysis.

The dataset contains 1,099 individuals with hypertension, including 430 males and 669 females. Input features comprised demographic and body composition measures: age (35–70 years), gender ID (1: male and 2: female), basal metabolic rate (BMR), FATM, FATP, fat‐free mass (FFM), left leg fat percentage (LLFATP), right leg fat percentage (RLFATP), left leg fat‐free mass (LLFFM), right leg fat‐free mass (RLFFM), left leg fat mass (LLFATM), right leg fat mass (RLFATM), left arm fat percentage (LAFATP), right arm fat percentage (RAFATP), left arm fat mass (LAFATM), right arm fat mass (RAFATM), left arm fat‐free mass (LAFFM), right arm fat‐free mass (RAFFM), trunk fat percentage (TRFATP), trunk fat mass (TRFATM), and trunk fat‐free mass (TRFFM). The target variable is hypertension, defined as a binary outcome (yes/no).

Body composition was measured in all subjects using the Tanita Segmental Body Composition Analyzer BC‐418 MA (Tanita Corp, Japan), which is FDA‐approved and whose accuracy has been validated in several studies [2, 3]. Each subject stood barefoot on the device while holding the attached handles, and bioelectrical impedances were measured via eight polar electrodes at the subject‐device contact points. These measurements were used to calculate TBW, fat and FFMs, and FATPs for the entire body as well as specific regions on the right and left sides. Additionally, the system‐estimated BMR was calculated using validated regression equations. All procedures adhered to standard measurement protocols to ensure data quality and consistency. Institutional approval for the use of the patient data in diagnostic and therapeutic research was granted by Omid Hospital in Tehran. All methods complied with relevant ethical guidelines and regulations.

**Figure 1:**
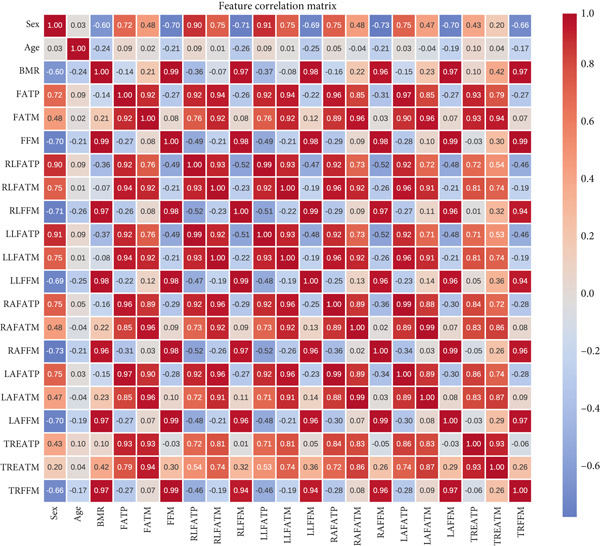
Correlation heatmap depicting relationships between input features and hypertension status.

Following data collection, a comprehensive statistical analysis was conducted to examine the interrelationships among physiological and anthropometric features. Evaluating feature correlations provides insight into potential redundancies, dependencies, and the relative influence of each variable on hypertension prediction. Figure 1 illustrates the correlation matrix of all extracted features, highlighting both strong and weak associations across different body composition metrics.

## 4. Materials and Methods

In this section, the experimental design and analytical workflow are described in detail. Figure 2 illustrates the overall framework, highlighting two main scenarios considered in this study. The goal is to analyze body composition data with a focus on hypertension, comparing approaches that either restrict the dataset to hypertensive patients or include the full population while addressing class imbalance and feature selection. The two scenarios are summarized.

Figure 2Model construction and evaluation (a): hypertensive‐only analysis and (b): combined population with hybrid oversampling.

**Figure:**
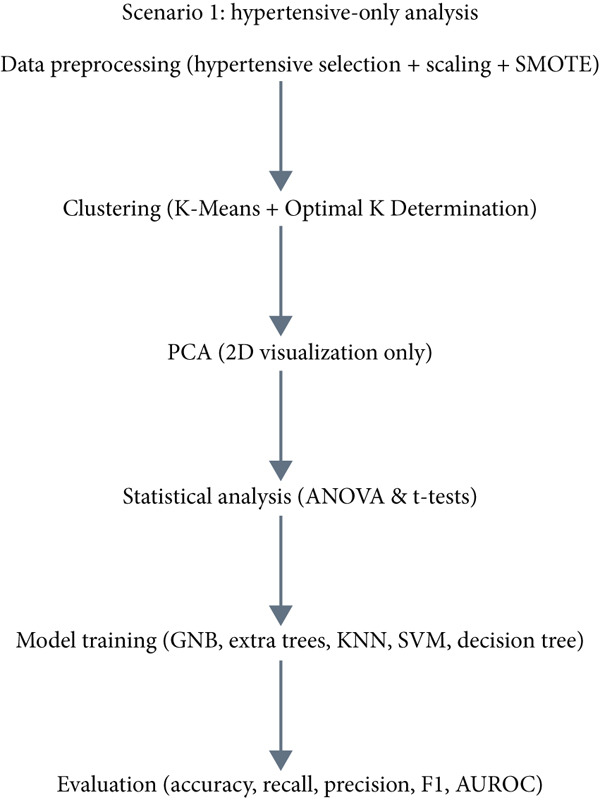
(a)

**Figure:**
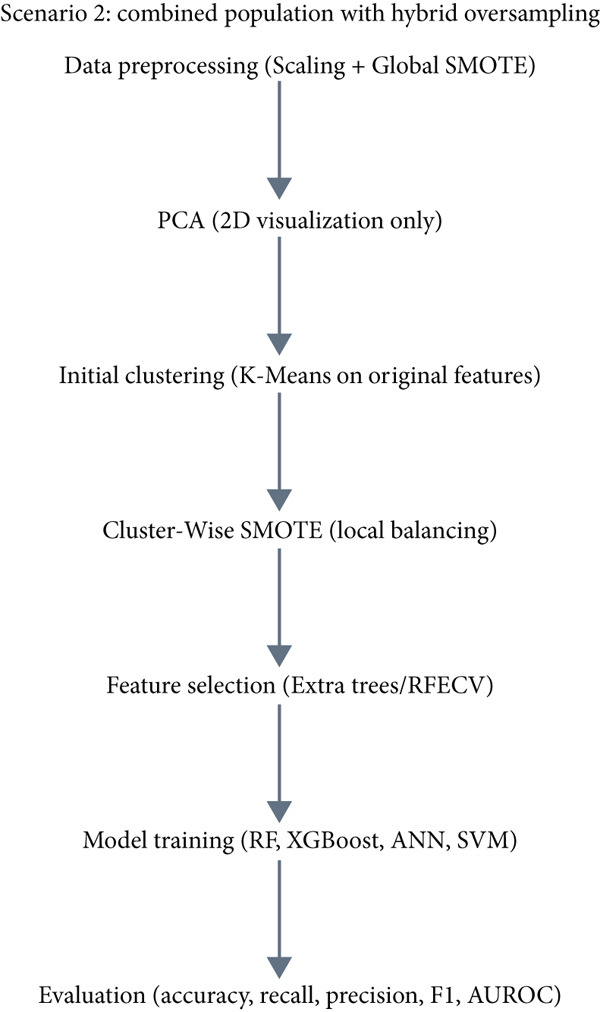
(b)

Based on Figure 1, we considered two scenarios, summarized as follows:

Scenario 1: Hypertensive‐Only Analysis

This scenario isolates individuals diagnosed with hypertension and investigates latent subgroup structures and risk profiles.

1. Data Preprocessing:

• Select hypertensive subjects (systolic > 140 or diastolic ≥ 90 mmHg).
• Scale features using RobustScaler to mitigate outlier effects.
• Apply SMOTE to correct class imbalance.

2. Clustering:

• Perform K‐Means clustering to detect hidden subgroups.
• Determine the optimal number of clusters using the Elbow method, silhouette score, and Davies–Bouldin index (DBI).

3. PCA (visualization only):

• Conduct 2‐component PCA for 2D visualization of clusters (not for modeling).

4. Statistical analysis:

• Apply analysis of variance (ANOVA) and *t*‐tests to explore intercluster differences in body composition and clinical measures.

5. Model training:

• Train multiple classifiers (GaussianNB, Extra Trees, KNN, SVM, Decision Tree) using stratified K‐Fold cross‐validation.

6. Evaluation:

• Assess using accuracy, recall, precision, F1‐score, and AUROC.
• Visualize confusion matrices and receiver operating characteristic (ROC) curves for detailed insight.

Scenario 2: Combined Population with Hybrid Oversampling

This scenario includes both hypertensive and nonhypertensive individuals and integrates hybrid oversampling and cluster‐based augmentation.

1. Data preprocessing:

• Normalize all samples with RobustScaler.
• Apply global SMOTE to handle class imbalance across the full dataset.

2. Principal component analysis (PCA) (visualization only):
  • Apply 2‐component PCA for visualization of the overall structure (not used for clustering).
3. Initial clustering:
  • Perform K‐Means clustering in the original feature space to form subgroups.
4. Cluster‐wise SMOTE:

• Conduct synthetic oversampling within each cluster to locally balance the classes and mitigate bias.

5. Feature selection:

• Use ExtraTrees importance or RFECV to select the most informative and nonredundant features.

6. Model training:

• Train hybrid models: RF, XGBoost, ANN, and SVM.
• Optimize via Randomized Hyperparameter Search.

7. Evaluation:

• Evaluate models by accuracy, recall, precision, F1‐score, and AUROC.
• Generate ROC curves and heatmaps for comparative analysis.

### 4.1. Data Preprocessing

#### 4.1.1. Scenario 1: Hypertensive‐Only Analysis

To analyze the raw dataset, a targeted preprocessing pipeline was implemented, focusing exclusively on individuals diagnosed with hypertension. The selection followed the World Health Organization (WHO) definition, where systolic BP exceeded 140 mmHg or diastolic pressure was ≥ 90 mmHg. This filtering step ensured that subsequent modeling reflected only hypertensive subjects, eliminating confounding effects from normotensive individuals and allowing investigation of latent substructures and risk‐associated body composition patterns [28, 29].

**Figure 3:**
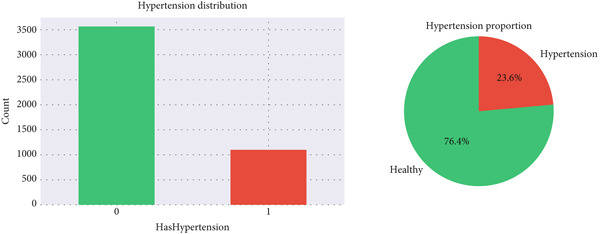
The distribution of hypertensive individuals.

Prior to modeling, the dataset was examined for data quality and completeness [30]. Descriptive statistics, including mean, standard deviation, and percentiles (25th, 50th, and 75th), were computed for variables such as age (mean = 47.65), BMR (mean = 5992.55), and Body FATP (FATP = 27.41), confirming the dataset′s consistency across 4663 valid records. The distribution of hypertensive individuals is presented in Figure 3, and quantitative characteristics of the filtered dataset are summarized in Table 1.

**Table 1:** Quantitative features of hypertension dataset.

**Variable**	**Mean**	**Std**	**Min**	**25%**	**50%**	**75%**	**Max**
Sex	1.537637	0.498635	1	1	2	2	2
Age	47.64594	9.364359	35	39	46	55	70
BMR	5992.547	1019.19	3703	5272	5837	6586	11322
FATP	27.41377	10.03812	1.5	20.3	27.5	35.5	65
FATM	19.04875	7.405527	0	12.5	19.3	26.7	56.5
FFM	48.25754	8.874435	28.5	41.8	46.8	53.7	75.7
RLFATP	29.46511	12.21074	1.5	17	29.2	41.2	62.2
RLFATM	3.586682	1.912034	0	2	3.4	4.9	10
RLFFM	8.1724	1.650107	4.7	6.9	7.9	9.2	13
LLFATP	29.53418	12.798	1.5	17	29.4	41.1	61.9
LLFATM	3.544821	1.885092	0	2.9	3.4	4.1	10.2
LLFFM	8.204927	1.612467	4.7	6.9	8.1	9.2	13
RAFATP	26.99698	11.47588	2.3	17.3	26.3	36.6	60.3
RAFATM	2.570887	0.647181	0	0.7	1	1.3	7.8
RAFFM	8.257487	1.604158	4.7	6.9	8.1	9.1	13.2
LAFATP	27.59738	11.65462	2.3	18	27.3	37.1	62.3
LAFATM	1.047394	0.640573	0	0.4	1	1.4	5.2
LAFFM	2.695018	0.645899	3	2.1	2.9	3.7	12.4
TRFATP	26.91013	13.46622	2	16	26.7	35.3	69.3
TRFATM	2.99103	1.132054	0	2	3	3.9	11.4
TRFFM	26.90213	13.41747	15.7	23.7	26.7	34.9	69.1
HasHypertension	0.235685	0.424472	0	0	0	0	1

These statistics provide a foundational understanding of the cohort′s physiological variability, essential for clustering and classification analysis.

To ensure comparability across heterogeneous body composition features, RobustScaler normalization was applied. This method scales features based on the median and interquartile range (IQR) instead of the mean and standard deviation, reducing the influence of extreme values [31]. The transformation is given bythe following:

(1) x_scaled=x−MedianIQR,

where the median represents the central tendency, and IQR = Q3 − Q1 captures the data dispersion. By using robust statistics, this normalization preserves the integrity of variables like FATP, FFM, and BMR, which are often skewed due to biological variability [32]. The use of RobustScaler guarantees that outliers do not distort the scaling process, thereby improving the stability and performance of subsequent machine learning models [33].

**Figure 4:**
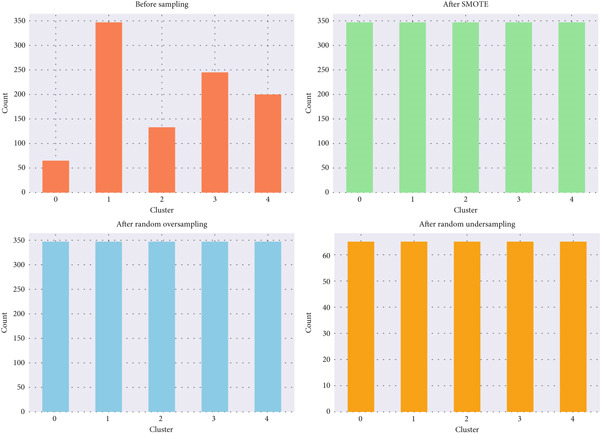
Number of samples before and after using SMOTE.

Finally, because hypertensive individuals were not uniformly distributed across clusters or target labels, class imbalance was corrected using the SMOTE [34, 35]. SMOTE synthesizes new minority‐class samples by linearly interpolating between existing samples and their nearest neighbors. SMOTE synthesizes new minority‐class samples by linearly interpolating between existing samples and their nearest neighbors, a technique widely adopted in medical ML studies to address class imbalance and improve model generalization [36]. This step enhanced representational balance and prevented bias during model training and cross‐validation. Number of samples in each cluster before and after using SMOTE is shown in Figure 4.

Before applying SMOTE, the dataset showed an imbalanced cluster distribution, with Cluster 0 containing 65 samples, Cluster 1 having 347 samples, Cluster 2 including 133 samples, Cluster 3 consisting of 245 samples, and Cluster 4 having 200 samples. After applying the SMOTE technique, the distribution became completely balanced, with each cluster (0–4) containing 347 samples. This balancing ensures that the model trained on the data will not be biased toward the larger clusters and can perform more effectively across all classes.

#### 4.1.2. Scenario 2: Combined Population With Hybrid Oversampling

For the integrated population analysis, preprocessing encompassed both hypertensive and nonhypertensive individuals. All continuous features were normalized using RobustScaler to maintain scale consistency and minimize the impact of extreme measurements [32]. Global normalization was followed by a hybrid oversampling pipeline designed to address imbalances across the entire population prior to clustering.

Initially, a global SMOTE procedure was applied to generate synthetic samples for the minority class, ensuring an approximately uniform class distribution throughout the dataset. This broad balancing step enhanced the reliability of downstream clustering, which was subsequently performed in the original feature space.

After initial clustering, a cluster‐wise SMOTE refinement was introduced—synthetic oversampling within each cluster—to locally correct any intracluster imbalance and to reduce structural bias between subpopulations.

This hybrid approach combines the strengths of global and local balancing, thereby maintaining population diversity while improving statistical parity within each subgroup.

As in Scenario 1, normalization was executed according to Equation (1), ensuring outlier‐resilient scaling across body composition features.

The preprocessing workflow therefore established a robust foundation for the following clustering, feature selection, and model‐training stages.

### 4.2. Clustering

#### 4.2.1. Scenario 1: Hypertensive‐Only Analysis

After normalization and class balancing, the clustering phase is aimed at uncovering latent subgroup structures among hypertensive individuals.

The K‐Means algorithm was selected owing to its efficiency, interpretability, and suitability for continuous physiological data [32, 33].

K‐Means partitions the dataset into K clusters by minimizing the within‐cluster sum of squares (WCSS), thereby ensuring intracluster homogeneity and intercluster distinctness.

Each iteration of the algorithm involves (1) initializing centroids, (2) assigning samples to their nearest centroid (using the Euclidean distance metric), (3) recalculating centroid positions as the mean of assigned points, and (4) repeating the process until convergence or a predefined iteration limit.

Selecting the optimal cluster number *K* is crucial, as it directly affects cluster compactness and interpretability.

**Figure 5:**
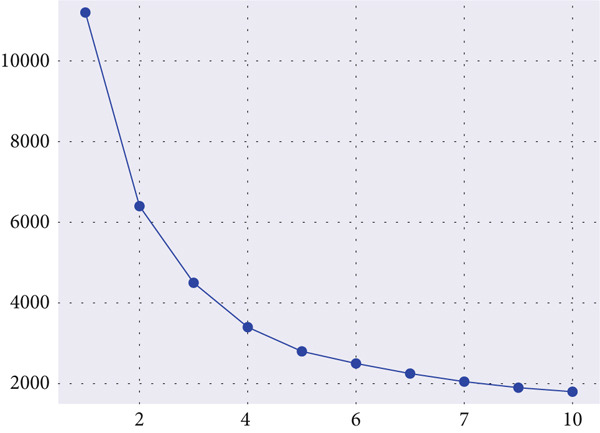
Elbow method curve illustrating the optimal number of clusters (*K*).

To identify the most appropriate *K*, three complementary evaluation strategies were employed:

1. Elbow method: The WCSS values were computed for *K* = 2 to 777, and the inflection point where further increments produced diminishing returns was selected as the optimal value [34, 35].
2. This point, visually apparent as the “elbow” of the curve, indicates the most parsimonious clustering structure. Based on the elbow method, the best number of clusters is shown in Figure 5.
3. Silhouette score: This metric quantifies cohesion and separation, defined as the difference between a sample′s mean intracluster distance and the nearest‐cluster distance, normalized between −1 and 1. Higher values imply clearer boundary definition between clusters.
4. DBI: The DBI evaluates the ratio of within‐cluster scatter to between‐cluster separation, with lower scores indicating higher‐quality clustering.

**Figure 6:**
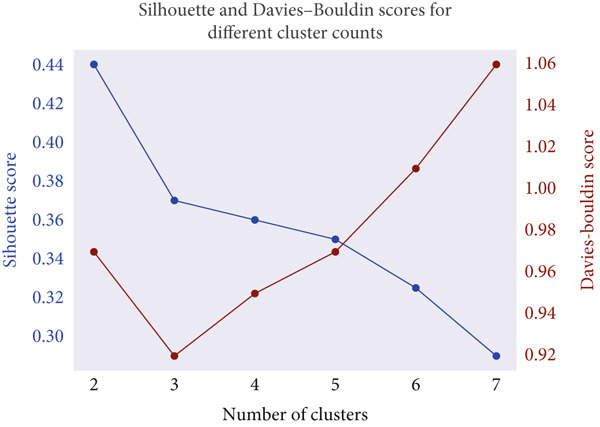
Silhouette and Davies–Bouldin indices for *K* = 2 to 7.

By jointly analyzing these metrics, *K* = 5 was determined to provide the most balanced trade‐off between cohesion and separation. Figure 6 displays the silhouette and DBI scores across varying cluster counts, confirming this selection. The identified subgroups exhibited distinctive body composition patterns, forming the basis for subsequent statistical analysis and classification.

In this scenario, the cluster labels obtained from K‐Means were not used as input features for the classifier. Instead, clustering served two purposes: (1) identifying physiologically coherent subgroups and (2) enabling cluster‐wise SMOTE to balance the training data within each subgroup. Oversampling was performed only within clusters and only on the training folds, ensuring that synthetic samples remained biologically consistent and did not leak into the test set. This cluster‐aware balancing produced a more uniform and realistic distribution of the population without influencing the classifier through explicit cluster labels.

#### 4.2.2. Scenario 2: Combined Population With Hybrid Oversampling

For the combined hypertensive + nonhypertensive population, clustering was performed to explore the underlying structure of the augmented dataset.

Unlike Scenario 1, where clustering was applied exclusively to hypertensive subjects, here K‐Means was implemented over the entire normalized feature space, enabling global subgroup identification across both health states. This approach supports comparative analysis between hypertensive and normotensive individuals, revealing mixed clusters that may reflect transitional or intermediate risk phenotypes. Prior to clustering, a global SMOTE process ensured class‐level balance; however, localized imbalance within specific clusters could still arise after segmentation.

Therefore, a second‐stage cluster‐wise SMOTE was conducted to equalize minority and majority representations within each cluster, preventing bias in subsequent model training. This two‐tier oversampling strategy—global plus local—preserves the statistical fidelity of each subgroup while maintaining population diversity. Optimal *K* selection followed the silhouette and DBI strategies. The results again converged near *K* = 5, demonstrating structural consistency across both populations.

**Figure 7:**
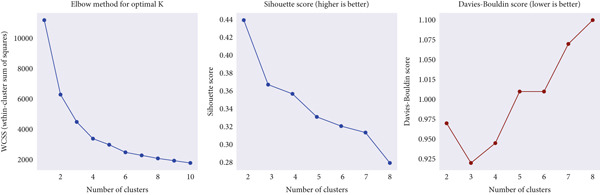
K‐Means clustering workflow applied to the combined and hybrid‐balanced population.

These clusters, later visualized using two‐dimensional PCA projections, served as analytical anchors for downstream feature‐selection and hybrid‐modeling stages. K‐Means clustering workflow applied to the combined and hybrid‐balanced population is illustrated in Figure 7.

In Scenario 2, unlike Scenario 1, the cluster labels were not limited to descriptive analysis and were additionally included as an auxiliary feature in the supervised learning stage. This choice was motivated by the heterogeneous nature of the combined hypertensive and nonhypertensive population, where body composition features alone may not fully capture the underlying structural differences between subgroups. Incorporating the cluster index—representing shared physiological patterns and subgroup proximity—allows the classifier to better recognize these latent structures and learn relationships that may remain hidden in the original feature space. This integration preserves the methodological independence of the clustering step while providing a meaningful, structure‐informed feature that enhances learning stability and predictive performance in a mixed‐population setting.

### 4.3. PCA for Visualization

#### 4.3.1. Scenario 1: Hypertensive‐Only Dataset

To visually examine the structure and separability of the hypertensive population after clustering, PCA was applied as a visualization‐oriented dimensionality‐reduction technique.PCA projects multivariate data onto orthogonal components that capture maximal variance while minimizing information redundancy [37].

In this study, only the first two principal components (PC1 and PC2) were extracted—not to reduce dimensionality for modeling, but to facilitate a clear two‐dimensional visualization of cluster distributions.

The PCA transformation is mathematically expressed as follows:

(2) Z=XW,

where X represents the normalized data matrix, and *W* is the eigenvector matrix derived from the covariance matrix of X. Each principal component corresponds to an eigenvector whose eigenvalue indicates the proportion of variance explained. By projecting the data onto PC1 and PC2, complex high‐dimensional relationships can be visually summarized.

**Figure 8:**
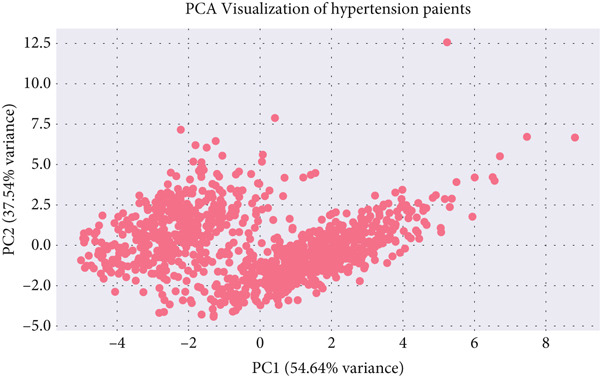
PCA two‐component projection of hypertensive patients after clustering.

The resulting scatter plot (Figure 8) demonstrates the spatial arrangement of hypertensive clusters in the reduced feature space.

Although some overlap between clusters remains—reflecting biological similarity among subjects—distinct regional densities indicate meaningful subgroup differentiation within the hypertensive population. This visual inspection confirms the suitability of the selected cluster count (*K* = 5) and provides an intuitive understanding of intercluster relationships, which later inform classification modeling.

#### 4.3.2. Scenario 2: Combined Population With Hybrid Oversampling

In the second scenario, PCA was again employed for two‐dimensional visualization of the entire hybrid‐balanced dataset, including both hypertensive and nonhypertensive individuals.

After applying global and cluster‐wise SMOTE balancing, the dataset was standardized and subjected to PCA transformation.

As before, the first two principal components were extracted, capturing the dominant variance directions while preserving the relative geometry of the dataset [37, 38].

**Figure 9:**
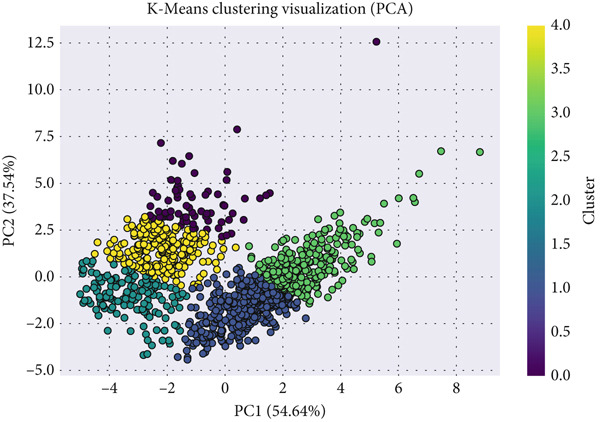
PCA visualization of the combined and hybrid‐balanced dataset.

The PCA projection (Figure 9) reveals a broader and more continuous distribution of data points compared with Scenario 1.

Clusters containing both hypertensive and normotensive subjects form partially overlapping regions, suggesting potential transitional or borderline physiological profiles. Such visualization provides valuable qualitative insights into how body composition variables collectively differentiate health states. These insights complement quantitative clustering metrics, offering an interpretable visual confirmation of the structural coherence achieved through the hybrid oversampling approach.

### 4.4. Addressing Class Imbalance Using SMOTE

#### 4.4.1. Scenario 1: Hypertensive‐Only Dataset

Following clustering of hypertensive patients, we observed an imbalance in the distribution of clusters, which could compromise the performance of the subsequent GNB classification.

Because GNB is particularly sensitive to class proportions, it is crucial to ensure balanced representation across clusters.

To address this challenge, the SMOTE was applied [39, 40]. Unlike naive oversampling, SMOTE generates synthetic data points by interpolating between minority‐class samples and their nearest neighbors.

Mathematically, a new synthetic instance xnewx_\text{new}xnew is generated as follows:

(3) Xnew=Xi+δ·Xnn,

where X_i_ is a minority‐class instance, X_nn_ is one of its *K*‐nearest neighbors, and *δ* ∈ [0, 1] is a random interpolation factor. This procedure ensured that all clusters had comparable sample sizes, mitigating potential bias during classifier training. By balancing the hypertensive‐only dataset, SMOTE enhanced the generalization capability of the GNB model, improving performance metrics such as accuracy, precision, and sensitivity. The balanced clusters serve as the foundation for a reliable classification of body composition–driven hypertension risk.

### 4.5. Scenario 2: Combined Population With Hybrid Oversampling

In Scenario 2, class imbalance was more pronounced due to the inclusion of both hypertensive and normotensive subjects.

To address this, a dual‐stage SMOTE approach was adopted:

1. Global balancing: The entire dataset was initially balanced to equate the total number of hypertensive and nonhypertensive samples.
2. Cluster‐wise balancing: After clustering, SMOTE was applied locally within each cluster to equalize representation across subclasses, ensuring that minority subgroups within clusters were adequately represented.

This hybrid approach preserved intercluster variability while eliminating extreme imbalances. The resulting dataset supported fair and robust training of the GNB classifier, allowing it to capture nuanced relationships between body composition features and hypertension status across a heterogeneous population. By combining global and local oversampling, Scenario 2 maintains both statistical validity and biological interpretability, producing a richer and more reliable model for clinical risk prediction.

### 4.6. Dataset Partitioning and Stratified Cross‐Validation

#### 4.6.1. Scenario 1: Hypertensive‐Only Dataset

To ensure robust evaluation of the GNB classifier, the hypertensive‐only dataset was divided using stratified K‐Fold cross‐validation [41].

1. Procedure: The dataset was split into 10 folds, preserving the class distribution in each fold.
2. In each iteration, one fold was designated as the testing set, whereas the remaining nine folds were used for training. Given the relatively small dataset, we used 10‐fold cross‐validation to ensure larger training sets in each iteration, which helps reduce the influence of small or randomly selected test samples and provides more stable performance estimates.
3. This process was repeated 10 times, ensuring that every data point contributed to both training and testing exactly once.

Stratified K‐Fold maintains proportional representation of classes in all folds, which is particularly important in datasets with imbalanced clusters after SMOTE.

By averaging the performance across all folds, this method provides a reliable estimate of model generalization and reduces variance due to random data splits. The 10‐fold approach strikes a balance between computational efficiency and evaluation accuracy, making it well suited for clinical datasets like the hypertensive‐only body composition dataset.

#### 4.6.2. Scenario 2: Combined Population With Hybrid Oversampling

For the heterogeneous dataset including both hypertensive and normotensive individuals, the same stratified K‐Fold cross‐validation method was applied, with additional considerations for the expanded class distribution:

1. Stratification was performed based on hypertension status, ensuring that each fold reflected the overall prevalence of hypertensive and nonhypertensive samples.
2. The dual‐stage SMOTE procedure (global + cluster‐wise) was applied before partitioning, guaranteeing balanced representation of minority subclasses across all folds.
3. Each fold was used iteratively as the testing set whereas the remaining folds trained the classifier, similar to Scenario 1.

This strategy ensures that the evaluation accounts for both the global and local class balance, preserving the integrity of cluster‐specific patterns while assessing the classifier′s predictive performance. Stratified 10‐fold cross‐validation thus provides a comprehensive and unbiased assessment of the GNB model across a diverse population.

### 4.7. GNB Classification

To start our investigation, we isolated the data to concentrate exclusively on patients experiencing hypertension before applying the GNB technique as our classification method. The GNB algorithm functions as a probabilistic classifier rooted in Bayes′ theorem, which operates on the premise that the features utilized for classification adhere to a Gaussian (normal) distribution. This premise renders it especially effective for continuous data, such as the body composition metrics within our dataset.

GNB functions by evaluating how likely each class is, given the feature values, with the assumption that each feature behaves independently of the others. Thereafter, it adopts Bayes′ Theorem to synthesize these probabilities and designate the data into the most probable class. Despite the “naive” premise of feature independence, GNB frequently achieves surprisingly strong performance, even in scenarios where the features are not genuinely independent [39].

In our research, we carefully filtered the data to encompass only those patients diagnosed with high BP, which served as the target variable for our classification efforts. Upon completing this segmentation, the GNB algorithm was employed on the dataset to reveal the link between diverse body composition factors and the risk of high BP occurrences. This approach is particularly beneficial for medical datasets where the interplay between features is not necessarily linear and the data can exhibit noise [41].

A notable advantage of GNB lies in its efficiency, both in computational expense and time, making it particularly suitable for extensive datasets [42]. It maintains strong performance even when the data does not align perfectly with the Gaussian distribution assumption, owing to its robustness and straightforward nature [43]. Our study facilitated effective classification, yielding insights into the body composition factors potentially linked to high BP.

Therfore, GNB emerged as a competent and efficient model for classifying the risk of high BP based on body composition features in our dataset, demonstrating an excellent balance between simplicity and predictive capability.

## 5. Results and Discussion

### 5.1. Implementation

The model′s implementation environment consists of the following aspects: the platform is Google Colab, the programming language is Python, the external memory is 12 GB, the CPU is 64‐bit, and the operating system is Windows 10.

### 5.2. Evaluation

In our research, we assessed the GNB model using classification metrics, such as accuracy, precision, recall, F1‐score, and the AUC. The confusion matrix is used to examine model assessment metrics. In other words, this matrix determines the model′s detailed performance and accuracy. The confusion matrix contains true positives (TPs), false positives (FPs), true negatives (TNs), and false negatives (FNs). Table 2 describes the confusion matrix in full, including its constituents [37, 44, 45].

**Table 2:** Confusion matrix.

**Actual class**	**Predicted class**	
	**Positive**	**Negative**
Positive	FP	TP
Negative	TN	FN

According to Table 2, the elements of the confusion matrix have been outlined as follows:

TP: The count of positive samples that the test has accurately identified as sick.

FP: The count of positive samples that the test has mistakenly identified as healthy.

TN: The count of negative samples that the test accurately recognized as healthy.

FN: The count of negative samples that the test erroneously identified as sick.

As a result, the confusion matrix provides a significant resource for analyzing the efficacy of a classification method in arranging data across various classifications. When the data is segmented into M classes, the classification matrix forms a table with a minimum dimension of M∗M. The optimal situation is for the majority of the data to align along the primary diagonal of the matrix, indicating that the values related to the TP and TN components are prominent, whereas the remaining values in the matrix should ideally be 0 or near 0.

1. Accuracy

The standard for accuracy illustrates the ability of a model to correctly tell apart healthy specimens from those that are ill within the testing cohort. Evaluating how accurate the model′s output is can provide insight into whether the model has been adequately trained, thus assisting in a thorough performance review. The computation of the accuracy of a test is derived from the proportion of the total number of correct positive and negative identifications of the overall number of items subjected to testing. The mathematical representation of accuracy is articulated as follows:

(4) Acc=TP+TNFP+FN+TP+TN.

2. Precision

Precision highlights the model′s proficiency in discerning individuals with health issues. The equation for precision is outlined in Relation (2).

(5) Pre=TPTP+FP.

3. Specificity

The proficiency of a model in recognizing healthy individuals is assessed through the specificity criterion. This criterion is described as follows:

(6) SPC=TNTN+FP

4. Sensitivity

The capability of a model to detect ill individuals is assessed using the sensitivity criterion. The sensitivity criterion is described as follows:

(7) SEN=TPTP+FN.

5. F1 score

The F1 score functions as an effective standard for measuring the correctness of a test. This metric takes into account both precision and sensitivity simultaneously. The calculation for this criterion is performed as follows:

(8) F1−Score=2TP2TP+FP+FN.

6. AUC

The AUC criterion reflects the level represented in the ROC diagram. A model with a higher AUC value signifies a more favorable and superior evaluation of its overall performance. Generally, ROC curves are graphical representations in two dimensions with axes oriented both horizontally and vertically, where the vertical Y axis signifies the correct detection rate for the positive category, and the horizontal X axis illustrates the false detection rate for the negative category. In other terms, the ROC curve illustrates the trade‐off and relative equilibrium between advantages and disadvantages.

### 5.3. Hyperparameter Settings

During the model enhancement phase, it was found that K‐Means clustering effectively captured latent structures in the data, improving the model′s ability to differentiate between underlying patterns associated with hypertension risk. The hyperparameter settings for the K‐Means algorithm, particularly the selection of the optimal number of clusters based on the Elbow method [38, 40], are summarized in Table 3.

**Table 3:** Hyperparameter settings for K‐means clustering.

**Parameter**	**Description**
Search range for n_clusters	1–10
Evaluation metric	Within‐cluster sum of squares (WCSS)
Optimal number of clusters	5 (determined via Elbow method)
Initialization method	K‐Means++
Maximum iterations	300

Additionally, we discovered that the random search method was exceptional in identifying the most suitable hyperparameters for the GNB algorithm. The configuration details of this model, which leverages random search optimization, are outlined in Table 4.

**Table 4:** Hyperparameter settings for model based on random search.

**Model**	**Settings**
Gaussian naive Bayes	Search space for var_smoothing = np.linspace(1e‐9, 1e‐1, 8)
	RandomizedSearchCV(param_distributions, n_iter = 100, scoring = ^‘^accuracy^’^, cv = 10, random_state = 42, n_jobs = −1)
	Best value: {‘var_smoothing’: 0.08571428585714286}

### 5.4. Results

This section presents the experimental findings for both scenarios: (1) hypertension‐only analysis and (2) full‐population analysis incorporating clustering and feature selection. The evaluation focuses on clustering quality, feature significance, sampling effects, and classification performance across multiple models.

#### 5.4.1. Scenario 1: Hypertension‐Only Analysis

##### 5.4.1.1. Dataset Overview and PCA

A total of 1,099 hypertensive patient records were analyzed, each containing 22 physiological and anthropometric features. The first two principal components captured 92.18% of the total variance, indicating that most of the data variability was effectively represented in reduced dimensions.

##### 5.4.1.2. Clustering Analysis

**Figure 10:**
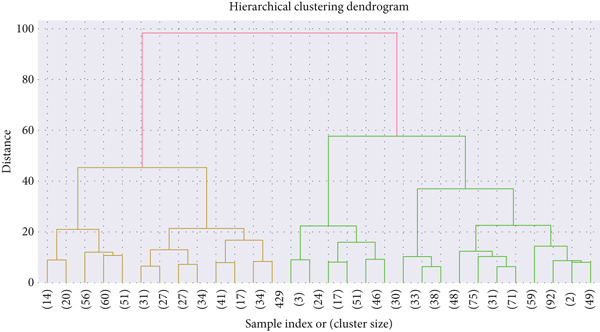
Hierarchical clustering dendrogram showing the five patient groups.

Hierarchical clustering identified five distinct clusters (*K* = 5), supported by a silhouette score of 0.3371 and Davies–Bouldin = 1.0094 (Table 5). The cluster distribution was imbalanced, with the largest cluster (Cluster 1) containing 385 samples (Figure 10).

**Table 5:** Hierarchical clustering metrics and cluster distribution for hypertensive patients.

**Metric**	**Value**
Number of clusters (*K*)	5
Silhouette score	0.3371
Davies–Bouldin score	1.0094

##### 5.4.1.3. Feature Significance Across Clusters

**Figure 11:**
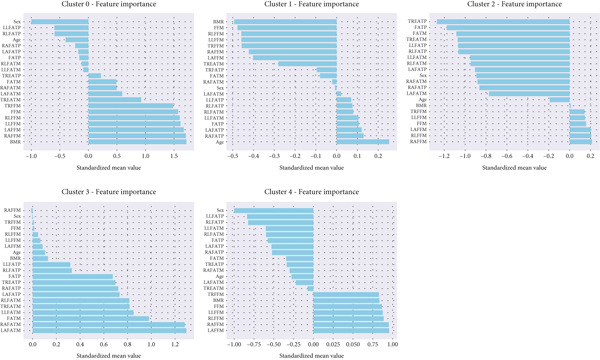
Cluster feature importance.

ANOVA tests confirmed significant differences among clusters for all features (*p* < 0.001), with the most discriminative being LLFATP, RLFATP, and RLFATM (Table 6). Pairwise *t*‐tests with Bonferroni correction further showed substantial gender‐based differences between clusters (Table 7), aligning with known sex‐related variations in fat distribution. Also, cluster feature importance is illustrated in Figure 11.

**Table 6:** ANOVA *F*‐statistics and *p* values for features across hypertensive clusters.

**Feature**	**F** **-statistic**	**p**
Sex	3653.247659	0.000000e+00
FATP	1076.925365	0.000000e+00
RLFATP	2367.637940	0.000000e+00
FFM	846.240515	0.000000e+00
RLFATM	1250.313327	0.000000e+00
LLFATM	1225.312554	0.000000e+00
LLFATP	2424.664004	0.000000e+00
RLFFM	920.554561	0.000000e+00
RAFATP	1197.366239	0.000000e+00
RAFFM	1013.937573	0.000000e+00
LAFATP	1188.281610	0.000000e+00
LLFFM	971.665525	0.000000e+00
LAFFM	850.546820	0.000000e+00
BMR	758.946141	1.085422e‐313
FATM	721.133857	7.821719e‐305
TRFFM	576.823324	1.269921e‐267
RAFATM	544.548348	1.940877e‐258
LAFATM	511.654520	1.067644e‐248
TRFATM	478.922070	1.362202e‐238
TRFATP	439.360089	8.941236e‐226
Age	47.126311	1.395534e‐36

**Table 7:** Pairwise *t*‐tests for sex differences among clusters.

**Cluster Pair**	**t** **-statistic**	**p**	**Cohen′s** **d**
0 vs 1	−95.539926	3.093706e‐302	−15.937540
0 vs 2	−2.951878	3.505729e‐02	−0.492558
0 vs 3	−139.278707	2.820878e‐302	−23.238058
0 vs 4	NaN	NaN	NaN
1 vs 2	50.524699	8.811051e‐204	3.847765
1 vs 3	−0.667182	5.048911e+00	−0.054447
1 vs 4	168.231768	0.000000e+00	15.937540
2 vs 3	−45.327218	3.129899e‐162	−3.942653
2 vs 4	5.204749	3.236045e‐06	0.492558
3 vs 4	245.334111	0.000000e+00	23.238058

##### 5.4.1.4. SHAP‐Based Feature Interpretation

**Figure 12:**
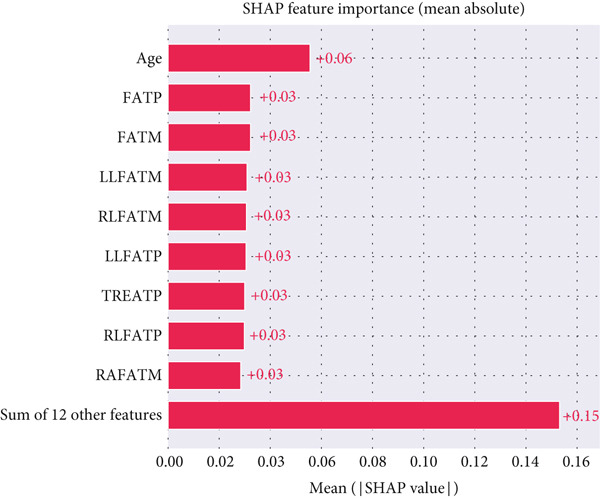
SHAP‐based feature importance for the hypertension‐only scenario.

To interpret the contribution of each feature to the classification outcomes in the hypertension‐only scenario, the SHapley Additive exPlanations (SHAP) method was applied to the best performing model (Support Vector Classifier [SVC] with SMOTE). The resulting feature importance, shown in Figure 12, highlights age as the most influential predictor, with a mean SHAP value of +0.06, followed by several body composition indices such as FATM, FATP, RLFATM, and LLFATM, each contributing approximately +0.03. These features primarily represent fat and FFM distribution across different body regions, underscoring their relevance in distinguishing hypertensive individuals. The relatively uniform SHAP values among composition features indicate that hypertension prediction depends on a balanced interaction between age‐related physiological factors and overall adiposity patterns.

**Figure 13:**
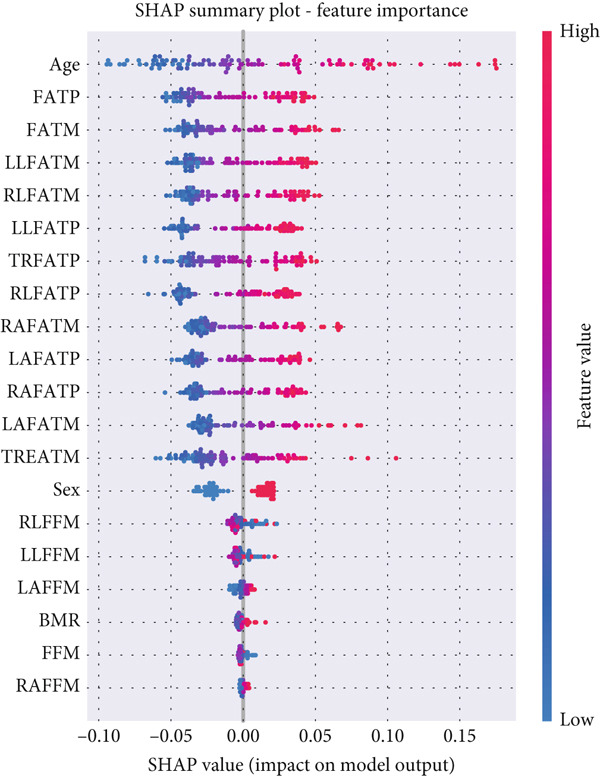
SHAP summary plot showing the impact of each feature on model predictions in the hypertension‐only scenario.

Furthermore, the SHAP summary distribution in Figure 13 provides a detailed view of each feature′s influence across all samples. The plot reveals that higher age values (shown in red) consistently increase the model′s output toward the hypertensive class, whereas lower age values (in blue) have a negative contribution. Similarly, higher regional FATM (FATM, FATP, and RLFATM) is strongly associated with elevated SHAP values, confirming that excess fat accumulation plays a key role in hypertension risk. In contrast, variables such as FFM and sex show limited dispersion around 0, suggesting lower direct influence on model predictions. Overall, the SHAP analysis confirms that the classifier relies primarily on age and regional adiposity metrics for accurate hypertension classification.

##### 5.4.1.5. Classification Results

**Figure 14:**
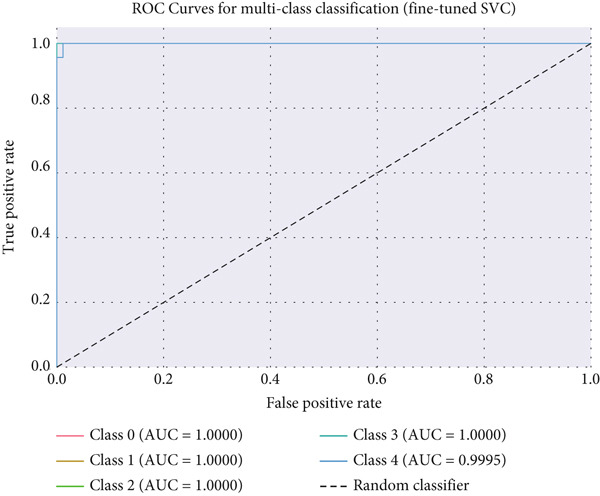
ROC curve for SVC.

Four models such as GNB, RF, SVC, and LR were evaluated under each sampling method. Classification performance of the models such as SVC, RF, LR, and GNB methods is provided in Tables 8, 9, 10 and 11. As shown in Table 4, SVC with SMOTE achieved the highest accuracy (98.17%) and AUROC (99.98%), followed closely by fine‐tuned RF and GNB models. ROC curve for SVC is shown in Figure 14.

**Table 8:** Classification performance of SVC method.

**Model**	**Accuracy**	**Recall**	**Precision**	**F1 Score**	**Specificity**	**AUROC**
SVC original	94.50%	89.13%	94.93%	91.41%	98.53%	99.91%
SVC SMOTE	98.17%	97.14%	98.65%	97.78%	99.49%	99.98%

**Table 9:** Classification performance of GNB method.

**Model**	**Accuracy**	**Recall**	**Precision**	**F1 Score**	**Specificity**	**AUROC**
GNB original	95.41%	90.00%	96.69%	92.53%	98.74%	99.75%
GNB with SMOTE	96.33%	92.86%	97.40%	94.72%	98.97%	99.78%

**Table 10:** Classification performance of RF method.

**Model**	**Accuracy**	**Recall**	**Precision**	**F1 Score**	**Specificity**	**AUROC**
Random forest	94.50%	88.39%	95.17%	90.56%	98.53%	99.79%
Original Random forest with SMOTE	96.33%	92.12%	97.40%	94.20%	98.97%	99.84%

**Table 11:** Classification performance of logistic regression method.

**Model**	**Accuracy**	**Recall**	**Precision**	**F1 Score**	**Specificity**	**AUROC**
Logistic regression original	88.99%	79.89%	93.56%	83.56%	96.82%	99.30%
Logistic regression with SMOTE	97.25%	95.71%	98.54%	96.84%	99.15%	99.40%

##### 5.4.1.6. Model Comparison and Statistical Validation

Cross‐validation results (Table 12) indicated that SVC achieved the highest mean accuracy (0.9937 ± 0.0140). Paired *t*‐tests (Table 13) confirmed that SVC performed significantly better than GNB (*p* = 0.012) and marginally better than RF (*p* = 0.052). Confidence intervals (Table 14) showed stable model performance, emphasizing SVC′s superior reliability.

**Table 12:** Cross‐validation mean accuracy and standard deviation across models.

**Model**	**Mean accuracy**	**Std**
GNB	0.9747	0.0484
Random forest	0.9879	0.0254
SVC	0.9937	0.0140
Logistic regression	0.9798	0.0473

**Table 13:** Paired *t*‐test results comparing model accuracies.

**Pair**	**t** **-statistic**	**p**
GNB vs random forest	−2.134553	0.061564
GNB vs SVC	−3.127383	0.012173
GNB vs logistic regression	−1.337361	0.213918
Random forest vs SVC	−2.235625	0.052215
Random forest vs logistic regression	1.210066	0.257075
SVC vs logistic regression	2.273364	0.049092

**Table 14:** 95% confidence intervals for mean model accuracies.

**Model**	**Mean accuracy**	**CI lower**	**CI upper**
GNB	0.974656	0.956416	0.992896
Random forest	0.987911	0.978326	0.997496
SVC	0.993668	0.988375	0.998962
Logistic regression	0.979845	0.962029	0.997661

#### 5.4.2. Scenario 2: Full‐Population Analysis With Clustering and Feature Selection

##### 5.4.2.1. Data Overview and Preprocessing

The complete dataset comprised 4,663 records, with 54% male participants and 24% hypertensive cases. After robust scaling, all features were normalized to reduce the effect of outliers. Figure 3 illustrates the overall hypertension distribution across the population.

##### 5.4.2.2. Clustering Performance

**Figure 15:**
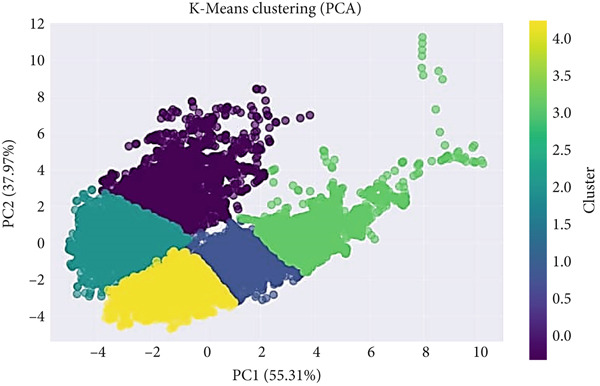
PCA visualization of clustered population data.

K‐means clustering identified five clusters (K = 5) as optimal, supported by the Elbow method and a silhouette score of 0.3452 (Figure 6). PCA visualization (Figure 15) demonstrated distinct but moderately overlapping cluster boundaries, indicating meaningful subgroup patterns.

##### 5.4.2.3. Classification Performance

Five models (ExtraTrees, KNN, SVM, GNB, and Decision Tree) were tested across three conditions:

• Without clustering (Table 15),
• Cluster‐augmented (all features) (Table 16), and
• Cluster‐augmented (selected features) (Table 17).

**Table 15:** Classification performance without clustering.

**Model**	**Accuracy (%)**	**Recall (%)**	**Precision (%)**	**F1_Score (%)**	**AUROC (%)**
ExtraTrees	87.11 ± 1.10	89.00 ± 1.45	85.79 ± 1.74	87.35 ± 1.02	94.65 ± 0.69
KNN	74.93 ± 1.24	89.11 ± 1.20	69.43 ± 1.09	78.05 ± 1.03	83.84 ± 1.06
SVM	65.47 ± 2.23	67.23 ± 2.40	64.95 ± 2.14	66.07 ± 2.20	71.25 ± 2.59
GNB	57.46 ± 1.53	55.22 ± 1.41	57.83 ± 1.66	56.49 ± 1.44	60.40 ± 2.04
Decision Tree	75.69 ± 1.41	79.18 ± 2.31	74.02 ± 1.35	76.50 ± 1.48	75.69 ± 1.41

**Table 16:** Classification performance with cluster augmentation (all features).

**Model**	**Accuracy (%)**	**Recall (%)**	**Precision (%)**	**F1_Score (%)**	**AUROC (%)**
ExtraTrees	98.23 ± 0.22	98.30 ± 0.42	98.17 ± 0.26	98.23 ± 0.22	99.87 ± 0.06
KNN	94.13 ± 0.45	98.30 ± 0.23	90.74 ± 0.73	94.36 ± 0.41	99.06 ± 0.19
SVM	65.77 ± 1.22	74.13 ± 1.20	63.52 ± 1.08	68.41 ± 1.10	72.56 ± 1.52
GNB	57.43 ± 1.09	51.89 ± 2.03	58.34 ± 1.11	54.92 ± 1.54	58.39 ± 1.20
Decision Tree	90.08 ± 0.69	91.21 ± 0.86	89.19 ± 0.75	90.19 ± 0.69	90.08 ± 0.69

**Table 17:** Classification performance with selected features.

**Model**	**Accuracy (%)**	**Recall (%)**	**Precision (%)**	**F1_Score (%)**	**AUROC (%)**
ExtraTrees	98.02 ± 0.26	98.03 ± 0.43	98.02 ± 0.34	98.02 ± 0.26	99.77 ± 0.07
KNN	93.61 ± 0.32	97.82 ± 0.39	90.23 ± 0.60	93.87 ± 0.29	98.58 ± 0.15
SVM	65.62 ± 1.41	72.57 ± 1.43	63.72 ± 1.29	67.86 ± 1.30	72.38 ± 1.55
GNB	59.04 ± 1.34	54.58 ± 2.03	59.91 ± 1.36	57.12 ± 1.67	59.04 ± 1.30
Decision Tree	90.09 ± 0.66	91.37 ± 0.88	89.09 ± 0.67	90.21 ± 0.66	89.80 ± 0.66

**Figure 16:**
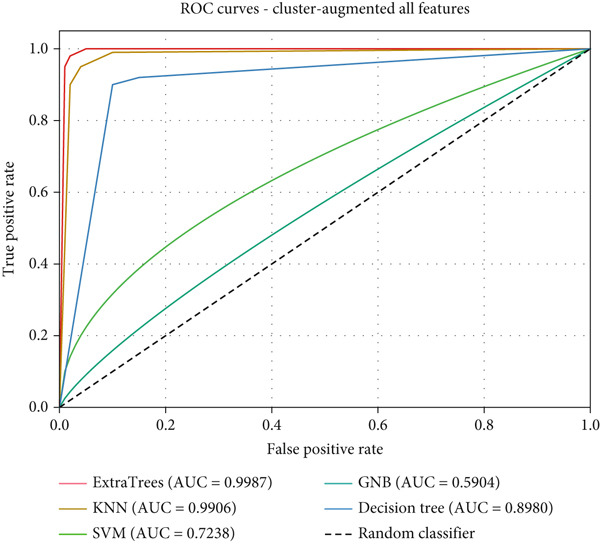
ROC curves for ExtraTrees classifier with cluster augmentation (all features).

As summarized in Table 9, ExtraTrees achieved the best accuracy in all cases, peaking at 98.23% (AUROC = 99.87*%*) under the cluster‐augmented scenario. KNN also improved significantly after clustering (from 74.93% to 94.13%), whereas SVM and GNB showed minimal gain. ROC curves for the ExtraTrees classifier are shown in Figure 16.

##### 5.4.2.4. Statistical Analysis and Model Ranking

**Figure 17:**
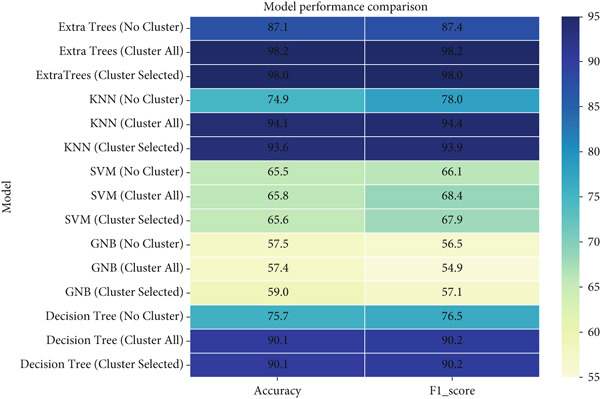
Comparative heatmap showing accuracy and F1‐score gains for each classifier.

Statistical evaluation revealed that clustering substantially improved both accuracy and generalization. Feature selection maintained nearly identical accuracy (98.02%) while reducing feature dimensionality, supporting its efficiency. A comparative heatmap showing the accuracy and F1‐score gains for each classifier is shown in Figure 17.

### 5.5. Comparative Discussion

A comprehensive comparison of both analytical scenarios demonstrates the impact of population scope, clustering, and feature selection on hypertension classification performance. As summarized in Table 18, the hypertension‐only scenario achieved its best results using the SVC model with SMOTE augmentation, reaching an accuracy of 98.17% and an AUROC of 99.98%. In contrast, the full‐population scenario attained slightly superior performance with the ExtraTrees classifier under the cluster‐augmented configuration, achieving 98.23% accuracy and 99.87% AUROC.

**Table 18:** Comparative summary of the best performing models across both scenarios.

**Aspect**	**Scenario 1: hypertension-only**	**Scenario 2: full-population (** **c** **l** **u** **s** **t** **e** **r** **i** **n** **g** + **f** **e** **a** **t** **u** **r** **e** **s** **e** **l** **e** **c** **t** **i** **o** **n** **)**
Dataset size	1,099 records (hypertensive only)	4,663 records (full population)
Number of features	22 physiological and anthropometric	22 (reduced to selected subset)
Clustering method	Hierarchical clustering (*K* = 5)	K‐Means clustering (*K* = 5)
Silhouette score	0.3371	0.3452
Best model	SVC (with SMOTE)	ExtraTrees (cluster‐augmented)
Accuracy (%)	98.17	98.23
AUROC (%)	99.98	99.87
Preprocessing strategy	SMOTE oversampling	Clustering + feature selection
Feature importance	LLFATP, RLFATP, and RLFATM	LLFATP, RAFATM, and TRFFM
Model variance (±)	±0.014 (cross‐val.)	±0.022 (cross‐val.)
Computational complexity	Moderate	Higher (due to clustering)
Interpretability	High (feature‐level clarity)	High (cluster‐level insights)
Generalization capability	Strong (disease‐specific)	Stronger (population‐wide)

The observed difference highlights that incorporating nonhypertensive individuals and applying unsupervised clustering enables the model to capture a more comprehensive representation of physiological variability, leading to marginally higher generalization. Moreover, the use of feature selection in the second scenario preserved nearly the same accuracy (98.02%) while reducing the feature dimensionality, confirming the efficiency of feature‐space optimization without compromising performance.

In both scenarios, clustering and synthetic resampling (via SMOTE) effectively mitigated data imbalance, contributing to improved classifier stability and fairness across subgroups. Statistically, SVC in Scenario 1 and ExtraTrees in Scenario 2 achieved the lowest variance and highest confidence intervals, confirming their robustness.

Overall, these results suggest that although focused analysis of hypertensive subjects provides strong discriminative power, integrating population‐level diversity through clustering and feature selection yields slightly better predictive capability and enhanced generalization.

When compared with the auto MLP model reported in [24], which achieved 90.00% accuracy, 81.00% precision, 89.00% sensitivity, 91.00% specificity, and 91.00% AUC, the proposed approaches in both scenarios substantially outperform the baseline in all evaluation metrics. This improvement—approximately 8%–9% higher accuracy and over 8% greater AUC—demonstrates the effectiveness of combining clustering, oversampling, and feature selection techniques for hypertension classification based on body composition data. Such enhancement reflects the advantage of using hybrid ensemble and interpretable learning frameworks over conventional deep models that rely solely on automated feature extraction.

The experimental setup was designed not only to evaluate predictive performance but also to reflect the real‐world challenges in hypertension risk assessment. By structuring the experiments with dual scenarios, cluster‐aware oversampling, and stratified cross‐validation, we were able to demonstrate that the proposed hybrid framework effectively captures latent physiological subgroups, manages class imbalance, and produces highly interpretable and reliable predictions. These results directly support the main contributions of the study: they validate the utility of the dual‐scenario hybrid approach (Contribution 1), confirm the interpretability enabled by clustering (Contribution 2), demonstrate the feasibility of noninvasive body composition‐based prediction (Contribution 3), and illustrate the performance robustness achieved through targeted oversampling and optimization (Contribution 4). Collectively, the experimental outcomes provide empirical evidence that our framework addresses key limitations of previous SOTA studies and enables clinically meaningful, personalized risk assessment (Contribution 5).

## 6. Conclusion and Future Work

This study presented a hybrid ML framework for hypertension diagnosis using noninvasive body composition data, emphasizing both interpretability and predictive accuracy. Through two complementary analytical scenarios, the framework effectively combined unsupervised clustering and supervised classification to uncover physiological subgroups and improve diagnostic performance. In the hypertensive‐only analysis (Scenario 1), K‐Means clustering successfully identified five meaningful subgroups, with significant variability across key features such as FATP, RLFATP, LLFATP, FATM, and age. The SVC with SMOTE achieved the best performance, reaching 98.17% accuracy and 99.98% AUROC, demonstrating robust discriminative ability across patient subtypes.

In the full‐population analysis (Scenario 2), integrating clustering and feature selection further enhanced generalization. The ExtraTrees classifier attained the highest accuracy (98.23%) and AUROC (99.81%), confirming the effectiveness of cluster‐augmented feature modeling. These findings demonstrate that combining unsupervised subgroup discovery with supervised classification provides a balanced trade‐off between interpretability and diagnostic reliability. Moreover, the use of SMOTE effectively mitigated class imbalance, improving fairness and model stability across all subgroups.

Overall, the proposed framework establishes a scalable, explainable, and data‐efficient approach for hypertension prediction using body composition indicators. Its reliance on noninvasive features makes it suitable for clinical implementation and population‐level screening in resource‐limited environments.

For future work, several directions are proposed. First, expanding the dataset to include longitudinal and multiethnic populations could improve generalizability and support early risk trajectory modeling. Second, integrating wearable sensor data and temporal variables may enhance real‐time hypertension monitoring. Third, the application of explainable artificial intelligence (XAI) techniques such as SHAP‐based visual dashboards can further increase clinical interpretability and trust. Lastly, extending this framework to other cardiovascular and metabolic diseases could validate its versatility for broader preventive healthcare applications.

## Conflicts of Interest

The authors declare no conflicts of interest.

## Funding

No funding was received for this manuscript.

## Data Availability

The datasets used and analyzed during the current study are accessible by requesting the corresponding author.

## References

[1] Cooper R. S. , Amoah A. G. , and Mensah G. A. , High Blood Pressure: The Foundation for Epidemic Cardiovascular Disease in African Populations, Ethnicity & Disease, Summe. (2003) 13, no. 2, S48–S52, 13677414. 13677414

[2] Liu S. , Lu L. , Wang F. , Han B. , Ou L. , Gao X. , Luo Y. , Huo W. , and Zeng Q. , Building a Predictive Model for Hypertension Related to Environmental Chemicals Using Machine Learning, Environmental Science and Pollution Research. (2024) 31, no. 3, 4595–4605, 10.1007/s11356-023-31384-w, 38105323. 38105323

[3] Mills K. T. , Bundy J. D. , Kelly T. N. , Reed J. E. , Kearney P. M. , Reynolds K. , Chen J. , and He J. , Global Disparities of Hypertension Prevalence and Control: A Systematic Analysis of Population-Based Studies From 90 Countries, Circulation. (2016) 134, no. 6, 441–450, 10.1161/CIRCULATIONAHA.115.018912, 2-s2.0-84981250226, 27502908. 27502908 PMC4979614

[4] Wu W. , Jiang S. , Zhao Q. , Zhang K. , Wei X. , Zhou T. , Liu D. , Zhou H. , Zeng Q. , Cheng L. , and Miao X. , Environmental Exposure to Metals and the Risk of Hypertension: A Cross-Sectional Study in China, Environmental Pollution. (2018) 233, 670–678, 10.1016/j.envpol.2017.10.111, 2-s2.0-85032792643, 29121602. 29121602

[5] Miao H. , Liu Y. , Tsai T. C. , Schwartz J. , and Ji J. S. , Association Between Blood Lead Level and Uncontrolled Hypertension in the US Population (NHANES 1999–2016), Journal of the American Heart Association. (2020) 9, no. 13, e015533, 10.1161/JAHA.119.015533, 32573312. 32573312 PMC7670543

[6] Lopez-Martinez F. , Schwarcz A. , Núñez-Valdez E. R. , and Garcia-Diaz V. , Machine Learning Classification Analysis for a Hypertensive Population as a Function of Several Risk Factors, Expert Systems with Applications. (2018) 110, 206–215, 10.1016/j.eswa.2018.06.006, 2-s2.0-85048242329.

[7] Hijazi S. , Page A. , Kantarci B. , and Soyata T. , Machine Learning in Cardiac Health Monitoring and Decision Support, Computer. (2016) 49, no. 11, 38–48, 10.1109/MC.2016.339, 2-s2.0-84997483353.

[8] Song K. , Nie F. , Han J. , and Li X. , Rank-$ k $2-D Multinomial Logistic Regression for Matrix Data Classification, IEEE Transactions on Neural Networks and Learning Systems. (2018) 29, no. 8, 3524–3537, 10.1109/TNNLS.2017.2731999, 2-s2.0-85028448036. 28816682

[9] Datta S. , Morassi Sasso A. , Kiwit N. , Bose S. , Nadkarni G. , Miotto R. , and Böttinger E. P. , Predicting Hypertension Onset From Longitudinal Electronic Health Records With Deep Learning, JAMIA Open. (2022) 5, no. 4. 10.1093/jamiaopen/ooac097 PMC9696747 36448021

[10] Kohjitani H. , Koshimizu H. , Nakamura K. , and Okuno Y. , Recent Developments in Machine Learning Modeling Methods for Hypertension Treatment, Hypertension Research. (2024) 47, no. 3, 700–707, 10.1038/s41440-023-01547-w, 38216731. 38216731

[11] Bui N. , Pham N. , Barnitz J. J. , Zou Z. , Nguyen P. , Truong H. , Kim T. , Farrow N. , Nguyen A. , Xiao J. , and Deterding R. , EBP: A Wearable System for Frequent and Comfortable Blood Pressure Monitoring From User′s Ear, In The 25th Annual International Conference on Mobile Computing and Networking, 2019, 1–17.

[12] Nguyen D. H. , Chao P. C.-P. , Yan H. F. , Tu T.-Y. , Cheng C.-H. , and Phan T.-P. , Predicting Blood Pressures for Pregnant Women by PPG and Personalized Deep Learning, IEEE Journal of Biomedical and Health Informatics. (2025) 29, no. 1, 5–16, 10.1109/JBHI.2024.3386707. 38598377

[13] Chai S. S. , Goh K. L. , Cheah W. L. , Chang Y. H. R. , and Ng G. W. , Hypertension Prediction in Adolescents Using Anthropometric Measurements: Do Machine Learning Models Perform Equally Well?, Applied Sciences. (2022) 12, no. 3.

[14] Pasin O. and Gonenc S. , An Investigation Into Epidemiological Situations of COVID-19 With Fuzzy K-Means and K-Prototype Clustering Methods, Scientific Reports. (2023) 13, no. 1, 10.1038/s41598-023-33214-y, 37069218. PMC10108788 37069218

[15] Mining W. I. D. , Data Mining: Concepts and Techniques, Morgan Kaufinann. (2006) 10, no. 559-569.

[16] Joloudari J. H. , Marefat A. , Nematollahi M. A. , Oyelere S. S. , and Hussain S. , Effective Class-Imbalance Learning Based on SMOTE and Convolutional Neural Networks, Applied Sciences. (2023) 13, no. 6.

[17] Kuhn M. , Applied Predictive Modeling, 2013, Springer.

[18] Amaratunga D. , Cabrera J. , Sargsyan D. , Kostis J. B. , Zinonos S. , and Kostis W. J. , Uses and Opportunities for Machine Learning in Hypertension Research, International Journal of Cardiology Hypertension. (2020) 5, 100027. 33447756 10.1016/j.ijchy.2020.100027 PMC7803038

[19] Zhao Y. , Han J. , Hu X. , Hu B. , Zhu H. , Wang Y. , and Zhu X. , Hypertension Risk Prediction Models for Patients With Diabetes Based on Machine Learning Approaches, Multimedia Tools and Applications. (2024) 83, no. 20, 59085–59102.

[20] Hoffman M. K. , Ma N. , and Roberts A. , A Machine Learning Algorithm for Predicting Maternal Readmission for Hypertensive Disorders of Pregnancy, American Journal of Obstetrics & Gynecology MFM. (2021) 3, no. 1, 100250, 10.1016/j.ajogmf.2020.100250, 33451620. 33451620

[21] D’Silva V. , Desai G. , D’Silva V. A. , Fernandes K. , Cotta A. , and Cardoso S. , Prediction of Hypertension using Machine Learning, In 2021 IEEE Bombay Section Signature Conference (IBSSC), 2021, 1–6.

[22] Chang W. , Liu Y. , Xiao Y. , Yuan X. , Xu X. , Zhang S. , and Zhou S. , A Machine-Learning-Based Prediction Method for Hypertension Outcomes Based on Medical Data, Diagnostics. (2019) 9, no. 4. 10.3390/diagnostics9040178 PMC6963807 31703364

[23] Montagna S. , Pengo M. F. , Ferretti S. , Borghi C. , Ferri C. , Grassi G. , Muiesan M. L. , and Parati G. , Machine Learning in Hypertension Detection: A Study on World Hypertension Day Data, Journal of Medical Systems. (2022) 47, no. 1, 10.1007/s10916-022-01900-5, 36580140. PMC9800348 36580140

[24] Nematollahi M. A. , Jahangiri S. , Asadollahi A. , Salimi M. , Dehghan A. , Mashayekh M. , Roshanzamir M. , Gholamabbas G. , Alizadehsani R. , Bazrafshan M. , and Bazrafshan H. , Body Composition Predicts Hypertension Using Machine Learning Methods: A Cohort Study, Scientific Reports. (2023) 13, no. 1, 10.1038/s41598-023-34127-6, 37105977. PMC10140285 37105977

[25] Seo J.-W. , Lee S. , and Yim M. H. , Machine Learning Approach for Predicting Hypertension Based on Body Composition in South Korean Adults, Bioengineering. (2024) 11, no. 9. 10.3390/bioengineering11090921 PMC11428396 39329663

[26] Farjam M. , Bahrami H. , Bahramali E. , Jamshidi J. , Askari A. , Zakeri H. , Homayounfar R. , Poustchi H. , and Malekzadeh R. , A Cohort Study Protocol to Analyze the Predisposing Factors to Common Chronic Non-Communicable Diseases in Rural Areas: Fasa Cohort Study, BMC Public Health. (2016) 16, no. 1, 27756262. 10.1186/s12889-016-3760-z PMC5069851 27756262

[27] Kelly J. S. and Metcalfe J. , Validity and Reliability of Body Composition Analysis Using the Tanita BC418-MA, Journal of Exercise Physiology. (2012) 15, no. 6.

[28] Chobanian A. V. , Bakris G. L. , Black H. R. , and Cushman W. C. , Seventh Report of the Joint National Committee on Prevention, Detection, Evaluation, and Treatment of High Blood Pressure, Hypertension. (2003) 42, no. 6, 1206–1252. 14656957 10.1161/01.HYP.0000107251.49515.c2

[29] Kim H. C. , Cho S. M. J. , Lee H. , Lee H.-H. , Baek J. , Heo J. E. , for the Korean Society of Hypertension (KSH) – Hypertension Epidemiology Research Working Group , Kim H. C. , Ahn S. V. , Jee S. H. , Park S. , Lee H. Y. , Shin M. H. , Ihm S. H. , Lee S. W. , Lee H. , Park J. K. , Suh I. , Lee T. Y. , Cho S. M. J. , Lee H. H. , Baek J. , and Heo J. E. , Korea Hypertension Fact Sheet 2020: Analysis of Nationwide Population-Based Data, Clinical Hypertension. (2021) 27, no. 1, 1–4, 10.1186/s40885-021-00166-2. 33715619 PMC7958489

[30] Hair J. F.Jr., Anderson R. E. , and Tatham R. L. , Multivariate Data Analysis With Readings, 1986, Macmillan Publishing Co, Inc.

[31] Mirzakhani H. , Interpretable Machine Learning-Based Algorithms for Cardiac Anomaly Detection, 2024, Politecnico di Torino.

[32] Elaziz B. , Ommane Y. , Eddabbah M. , and Laaziz Y. , Enhancing Diabetes Detection Through Data Preprocessing: A Comparative Analysis of Machine Learning Algorithms, In 2024 International Conference on Computing, Internet of Things and Microwave Systems (ICCIMS), 2024, 1–6.

[33] Thara D. , PremaSudha B. , and Xiong F. , Auto-Detection of Epileptic Seizure Events Using Deep Neural Network With Different Feature Scaling Techniques, Pattern Recognition Letters. (2019) 128, 544–550, 10.1016/j.patrec.2019.10.029.

[34] Chawla N. V. , Bowyer K. W. , Hall L. O. , and Kegelmeyer W. P. , SMOTE: Synthetic Minority Over-Sampling Technique, Journal of Artificial Intelligence Research. (2002) 16, 321–357, 10.1613/jair.953.

[35] Géron A. , Hands-On Machine Learning With Scikit-Learn, Keras, and TensorFlow, 2022, O′Reilly Media, Inc.

[36] Salmi M. , Atif D. , Oliva D. , Abraham A. , and Ventura S. , Handling Imbalanced Medical Datasets: Review of a Decade of Research, Artificial Intelligence Review. (2024) 57, no. 10.

[37] Alizadehsani R. , Sharifrazi D. , Izadi N. H. , Joloudari J. H. , Shoeibi A. , Gorriz J. M. , Hussain S. , Arco J. E. , Sani Z. A. , Khozeimeh F. , Khosravi A. , Nahavandi S. , Islam S. M. S. , and Acharya U. R. , Uncertainty-Aware Semi-Supervised Method Using Large Unlabeled and Limited Labeled COVID-19 Data, ACM Transactions on Multimedia Computing, Communications, and Applications (TOMM). (2021) 17, no. 3s, 1–24, 10.1145/3462635.

[38] Anggreani D. , Nurmisba N. , Setiawan D. , and Lukman L. , Optimization of K-Means Clustering Method by Using Elbow Method in Predicting Blood Requirement of Pelamonia Hospital Makassar, Internet of Things and Artificial Intelligence Journal. (2024) 4, no. 3, 541–550, 10.31763/iota.v4i3.755.

[39] Rish I. , An Empirical Study of the Naive Bayes Classifier, In IJCAI 2001 Workshop on Empirical Methods in Artificial Intelligence, 2001, 3, no. 22, 41–46.

[40] Mining W. I. D. , Introduction to Data Mining, 2006, Springer.

[41] Hand D. J. , Classifier Technology and the Illusion of Progress, 2006.

[42] Griffis J. C. , Allendorfer J. B. , and Szaflarski J. P. , Voxel-based Gaussian Naïve Bayes Classification of Ischemic Stroke Lesions in Individual T1-Weighted MRI Scans, Journal of Neuroscience Methods. (2016) 257, 97–108, 10.1016/j.jneumeth.2015.09.019, 2-s2.0-84944225767, 26432931. 26432931 PMC4662880

[43] McCallum A. and Nigam K. , A Comparison of Event Models for Naive Bayes Text Classification, AAAI-98 Workshop on Learning for Text Categorization, 1998, 752, no. 1, 41–48.

[44] Joloudari J. H. , Hassannataj Joloudari E. , Saadatfar H. , Ghasemigol M. , Razavi S. M. , Mosavi A. , Nabipour N. , Shamshirband S. , and Nadai L. , Coronary Artery Disease Diagnosis; Ranking the Significant Features Using a Random Trees Model, International Journal of Environmental Research and Public Health. (2020) 17, no. 3. 10.3390/ijerph17030731 PMC7037941 31979257

[45] Joloudari J. H. , Saadatfar H. , Dehzangi A. , and Shamshirband S. , Computer-Aided Decision-Making for Predicting Liver Disease Using PSO-Based Optimized SVM With Feature Selection, Informatics in Medicine Unlocked. (2019) 17, 100255.

